# Supplementary material for: Experimental and molecular predictions of the adjuvanticity of snail mucin on hepatitis B vaccine in albino mice
Source: PLoS One. 2021 Jul 23;16(7):e0246915. doi: 10.1371/journal.pone.0246915 (PMC8301616; doi:10.1371/journal.pone.0246915)
Supplement: S1 Table — (PDF) [file pone.0246915.s001.pdf]

S1 Table Effect of Hepatitis-B Vaccine Modulated with Egg Shell Membrane on Eosinophil Count

	**Eosinophil count (%) (Comparison within and across groups)**			
	**Day 0**	**Day 14**	**Day 21**	**Day 28**
**Group 1**	**0.00 ± 0.00 ^{aA}**	**0.00 ± 0.00 ^{aA}**	**0.00 ± 0.00^{aA}**	**0.33 ± 0.03 ^{aA}**
**Group 2**	**0.00 ± 0.00 ^{A}**	**0.00 ± 0.00 ^{A}**	**0.00 ± 0.00^{A}**	**0.00 ± 0.00 ^{A}**
**Group 3**	**1.67 ± 0.20 ^{aA}**	**1.33 ± 0.15 ^{aA}**	**1.33 ± 0.15^{aB}**	**1.00 ± 0.48 ^{aA}**
**Group 4**	**0.67 ± 0.17 ^{aA}**	**0.33 ± 0.03 ^{aA}**	**0.00 ± 0.00^{aA}**	**0.00 ± 0.00 ^{aA}**
**Group 5**	**0.00 ± 0.00 ^{aA}**	**0.00 ± 0.00 ^{aA}**	**0.33 ± 0.03^{aAB}**	**0.00 ± 0.00 ^{aA}**

Results are expressed in Means ± SD (n = 3)

Results are expressed as mean ± SD (n=3). Mean values with different small letters as superscripts within the groups were considered significant at $p < 0.05$ and mean values with different capital letters as superscripts across the groups were considered significant at $p < 0.05$

Group 1: rHBsAg vaccine alone: 3 doses

Group 2: rHBsAg vaccine alone: 2 doses

Group 3: rHBsAg vaccine + Snail mucin: 2 doses

Group 4: Snail mucin alone: 2 doses

Group 5: Normal saline: 3 doses
