# Supplementary material for: Experimental and molecular predictions of the adjuvanticity of snail mucin on hepatitis B vaccine in albino mice
Source: PLoS One. 2021 Jul 23;16(7):e0246915. doi: 10.1371/journal.pone.0246915 (PMC8301616; doi:10.1371/journal.pone.0246915)
Supplement: S3 Table — (PDF) [file pone.0246915.s003.pdf]

S3 Table: Effect of snail mucin-adjuvanted rHBsAg vaccine on basophil count of experimental mice

	Basophil count (%)				
	**Group 1**	**Group 2**	**Group 3**	**Group 4**	**Group 5**
**Day 0**	**0.00 ± 0.00**	**0.00 ± 0.00**	**0.00 ± 0.00**	**0.00 ± 0.00**	**0.00 ± 0.00**
**Day 14**	**0.00 ± 0.00**	**0.00 ± 0.00**	**0.00 ± 0.00**	**0.00 ± 0.00**	**0.00 ± 0.00**
**Day 21**	**0.00 ± 0.00**	**0.00 ± 0.00**	**0.00 ± 0.00**	**0.00 ± 0.00**	**0.00 ± 0.00**
**Day 28**	**0.00 ± 0.00**	**0.00 ± 0.00**	**0.00 ± 0.00**	**0.00 ± 0.00**	**0.00 ± 0.00**

Results are expressed as mean ± SD (n=3).

Group 1: rHBsAg vaccine alone: 3 doses

Group 2: rHBsAg vaccine alone: 2 doses

Group 3: rHBsAg vaccine + Snail mucin: 2 doses

Group 4: Snail mucin alone: 2 doses

Group 5: Normal saline: 3 doses
