# Supplementary material for: Experimental and molecular predictions of the adjuvanticity of snail mucin on hepatitis B vaccine in albino mice
Source: PLoS One. 2021 Jul 23;16(7):e0246915. doi: 10.1371/journal.pone.0246915 (PMC8301616; doi:10.1371/journal.pone.0246915)
Supplement: Attachment — Submitted filename: Response to Reviewer.pdf [file pone.0246915.s004.pdf]

// University of Nigeria, Nsukka
Faculty of Biological Sciences
Department of Biochemistry

Timothy P. C. Ezeorba

(Lecturer II)

24th May 2021

Dr. Isabelle Chemin,

Academic Editor,

PLOS ONE Journal.

Dear Dr. Isabelle,

RESPONSE TO ACADEMIC EDITOR AND REVIEWER

I sincerely appreciate the privilege of reviewing our research article entitled "**Experimental and Molecular Predictions of the Adjuvanticity of Snail Mucin on Hepatitis B Vaccine in Albino Mice**". Thank you very much for your comments and feedback, and also that of the reviewer. I have thoroughly effected all possible corrections and made relevant additions based on your feedback. I sincerely hope you find them meritorious for your reputable Journal (PLOS ONE).

Based on the Academic Editor comments which focused on the journal requirements, here are my responses and corrections that have been effected on the manuscripts

1. I ensured that the manuscript now meets the PLOS ONE's style requirements, especially with regards to proper figure and table labelling, using the right font size for level 1 and 2 headings and using upper cases only for the first letter of all headings.
2. In my first submission, I erroneously omitted the volume of blood samples used for the study in the method section. However, in this revised manuscript, the volume of blood collected, which was 0.5 mL, has been included in the method section.
3. Finally, the methods on handling the animals have been explicitly included in the revised manuscript. The method of **cervical dislocation** was adopted for animal euthanasia by a well-trained researcher, who ensured that the suffering of the animals were reduced to the barest minimum. We did not administer anaesthesia or analgesia to the animals despite

their pain-relieving activities, to avoid interference with other intended histopathology analysis. More so, snail mucin has been reported to possess some anaegestic properties, hence possibly reducing the pain in the animals (Adikwu and Nnamani, 2005).

Based on the peer-reviewer's major concerns, here are my responses and corrections that have been effected on the manuscripts.

Major concerns 1 and 2:

I sincerely appreciate all the comments of the reviewer.

Fig 4 from my initial submission was clumsy. It was a composite graph showing the IgG, IgG1 and IgG2 titre values together, for different time point. Hence, the figure did not explicitly despite some of the claims from my results.

To address this issue based on the reviewer suggestion, I have separated the composite graphs into three separate graphs. The new graphs (Fig 4a, 4b and 4c) now show the mean titre value for IgG, IgG1 and IgG2 with their various statistics (error bar) respectively.

The reviewer pointed out that my claim of observing a significantly ($P<0.05$) higher mean titre of anti-HBsAg bodies in group 3 (administered with mucin and rHBsAg) when compared to negative control (group 5) was correct. However, my result didn't support the assertion of a high antibody titre in group 3 when compared to the positive control (group 2-adminstered with rHBsAg only) at any time point.

The new Fig 4a showed that on day 28, a significantly higher IgG titre value of the experimental group 3 (administered with 2 doses of the vaccine and mucin), when compared with both the positive control group 2 (administered only with 2 doses of rHBsAg vaccine only) and the negative control group 4 and 5 (administered with only mucin and normal saline respectively).

Although, the result on day 14 and 21 did not show significant ($P>0.05$) differences in the IgG titre across the treatment group 3 and control group 2 & 5, as compared to what was observed on day 28. This result patterned our thinking to the possible mechanisms of adjuvanticity of snail mucin on rHBsAg, as was explained in the discussion section.

We explained that the mucin, possibly acts as a Polymeric Biodegradable Microparticles, entrapping the antigen and releasing it gradually over a period of time. The proposed antigen entrapment mechanism was supported by a marked significant ($p>0.05$) increase in the mean IgG

titre of the group 3 (co-administered snail mucin and vaccine) compared to the group 2 (administered 2 doses of the vaccine alone) on day 28 (Fig 4a).

Similarly, the new Fig 4b, showed a very interesting result. Although the IgG1 titre of the treatment group 3 was significantly lower than the positive control group 2 on day 21 and non-significantly lower on day 28, **there was a relatively significant boost in the IgG1 titre for the treatment group from day 21 to day 28, while the positive control group 2 showed a decrease in IgG1 titre from day 21 to day 28.** This further support our assumptions of possible antigen entrapping mechanism of Snail Mucin.

Lastly, the results of the new Fig 4c showed a contrasting trend for the mean titre value of IgG2. Studies have shown that Cytotoxic T-lymphocytes (CTLs) stimulated by Th1 response is not essential for protective immunity against HBV (Konda et al., 2013). It is known that IgG2a stimulates Th1 responses while IgG1 stimulates Th2 responses. Hence, the possible explanation for a low IgG2 titre across the group.

Major concerns 3:

> *The reviewer pointed out the need for toxicity studies which is very relevant for determining the effective dosage that can be accommodated in the animals.*

In this present study, it is true we did not perform any toxicity studies on the mice, despite its significance. However, our choice for the dose formulation for snail mucin was not done blindly but was guided by a study conducted by Adikwu and Nnamani (2005). It was reported in that study, that snail mucin was well tolerated, as the animals (mice 20-25 g) did not show any symptoms of toxicity and none of the animals died (Adikwu and Nnamani, 2005). Since, a similar experiment on the animals has been published in the past, we decided not to repeat the same experiments, with the aim of avoiding the unjustifiable use of animals for research - based on our institutional animal laws.

Moreover, our results from our study showed that snail mucin was non-toxic to the animals as, no animal died during the study, no significant changes in the weight of the animal and no obvious allergy reaction was observed at the point of administration. Finally, the liver weight and histology were similar for all the experimental groups.

Major concerns 4:

The reviewer recommended that a ***survival test study*** *be conducted to fully support the conclusions that snail mucin is helpful as an adjuvant for rHBsAg vaccine.*

It is true that we did not conduct a survival test, during the experimental phase of the studies. This study is relevant as it may give more confirmatory evidence to our assertion that snail mucin has some adjuvant properties in rHBsAg. However, our conclusion was based on general scientific knowledge about the correlation of elevated antibody titres with vaccine efficacy. Moreover, the survival test is imperative to determine whether these antibodies generated could really neutralize the virus.

The results we have presented have shown that snail mucin has some possible adjuvant properties, through the possible Polymeric Biodegradable entrapment mechanism – entrapping the antigen, releasing it in bits to sustained last longing immunity, and reducing the need for multiple vaccinations.

Despite the relevance of this suggested study (survival test), it may be very difficult to perform this experiment now as we have euthanised all the animals used for the studies. More so, culturing HBV for infecting the animal is very difficult due to restrictions within the institution when working with infectious viral particles. Regardless, this will be done in a future study, when we secure collaborations with a virology institute/laboratory.

Thank you very much for taking the time to go through my responses. Hope you find them worthy.

Your sincerely,

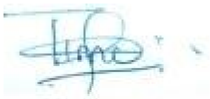

Timothy, P. C. Ezeorba

Timothy.ezeorba@unn.edu.ng
