# Supplementary material for: Experimental and molecular predictions of the adjuvanticity of snail mucin on hepatitis B vaccine in albino mice
Source: PLoS One. 2021 Jul 23;16(7):e0246915. doi: 10.1371/journal.pone.0246915 (PMC8301616; doi:10.1371/journal.pone.0246915)
Supplement: Attachment — Submitted filename: Rebuttal letter.pdf [file pone.0246915.s005.pdf]

University of Nigeria, Nsukka

Faculty of Biological Sciences

Department of Biochemistry

Timothy P. C. Ezeorba 24th May 2021

(Lecturer II)

Dr. Isabelle Chemin,

Academic Editor,

PLOS ONE Journal.

Dear Dr. Isabelle,

RESPONSE TO ACADEMIC EDITOR AND REVIEWER

Once again, I sincerely appreciate the privilege of reviewing our research article entitled "**Experimental and Molecular Predictions of the Adjuvanticity of Snail Mucin on Hepatitis B Vaccine in Albino Mice**". Thank you very much for your comments and feedback, and also that of the reviewer. I sincerely hope you find this rebuttal meritorious for your reputable Journal (PLOS ONE).

Review based on the journal requirements

> ***Please review your reference list to ensure that it is complete and correct. If you have cited papers that have been retracted, please include the rationale for doing so in the manuscript text, or remove these references and replace them with relevant current references. Any changes to the reference list should be mentioned in the rebuttal letter that accompanies your revised manuscript. If you need to cite a retracted article, indicate the article's retracted status in the References list and also include a citation and full reference for the retraction notice.***

I have reviewed the reference list and ensured that they are now appropriate. I replaced Ref number [11], Adikwu and Ikejiuba, (2005) with a new reference (Kalu et al., 2019).

Reason - I discovered that the journal website where Adikwu and Ikejiuba, (2005) published have been pulled out and is no longer accessible. The new reference (Kalu et al., 2019) is more recent and addresses similar methods and ideas as the previous reference used.

To the best of my knowledge, other references are appropriate for publication.

Based on the peer-reviewer's major concerns, here are my responses and corrections that have been effected on the manuscripts.

I want to sincerely appreciate the reviewer for the detailed and thorough review of this work. I have ensured to respond appropriate to the reviewer's comments to the best of my ability. Presented below are the reviewer's comments and my responses.

- Can the authors explain why Group 2 is similar to Group 4 (Snail Mucin only, no vaccine) on Day 28 in Fig.4a?

Thank you very much for pointing that out from our result as depicted in Figure 4a.

The results on Figure 4a show the IgG titre value measured using iELISA for all the experimental groups from day 0 to 28. The results on day 28 showed a similar IgG titre value for animals in group 2 (administered 2 doses of the vaccine alone) and group 4 (a negative control administered 2 doses of mucin alone), as pointed out by the reviewer. Intuitively, one may think that there should be a significant difference among the two groups, however, a non-significant ($P>0.05$) increase in group 2 as compared to group 4 was observed. The possible reasons for an increased IgG in the group 4 animals may be due to immunogenic responses in some of the animals within the group which may have been challenged with antigens within their environment (inhaled or ingested). This may be evident as pointed out by statistical analysis which showed a considerably higher error bar in group 4 on day 28, when compared to other experimental groups.

Furthermore, there are possibilities that snail mucin, since foreign to the animal, may have resulted in a level of antibody production, accounting for the IgG titre in group 4. Although there are very limited studies on the immunogenicity of snail mucins, few studies available highlighted contrasting opinions. A study conducted by Karne et al. (2017) showed mucin protein from ***Macrochlymus indica*** (a species of Terrestrial Land Snail) to possess some immunogenic properties after an IELISA experiment. While, another study conducted by Harti et al. (2018)

pointed out that snail mucus from ***Achatina fulica*** on its own could not stimulate lymphocyte proliferation. Hence, there is need for future studies to investigate thoroughly the immunogenic potential of Snail mucin from different varieties of snails.

- A figure legend usually has a title, and Materials and method (A description of the techniques used). P-values and the sample size, if applicable, should also be included. Please complete all figure legends in the manuscript.

Thank you very much for the comments.

It is very true that a description for each of the figures are necessary. I have done that in my previous submission. The figure legends were place in the body of the article, at a position where the Figures should be placed. This was in line with the journal format for submission.

However, I have improved the legends, by adding some of its vital components as you pointed out from your comment. Thank you.

Thank you very much for taking the time to go through my responses. Hope you find them worthy.

Your sincerely,

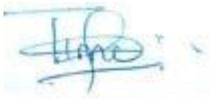

Timothy, P. C. Ezeorba

Timothy.ezeorba@unn.edu.ng

References

Karne S. P., Gupta A., Sumesh S., kamble, S. and Bharat S. (2017). Exploration of mucin protein from *Macrochlymus indica* and determined its immunogenicity studies. *Journal of Biomedical and Pharmaceutical Research*, *6*(1): 33-38. Retrieved from https://jbpr.in/index.php/jbpr/article/view/83

Harti, A. S., A. Murharyati, D. S. S, and M. Oktariani (2018). The effectiveness of snail mucus (*Achatina fulica*) and chitosan toward limfosit proliferation in vitro. *Asian Journal of Pharmaceutical and Clinical Research* 11(15): 85-88, doi:10.22159/ajpcr.2018.v11s3.30041
